# ‘They treat us like machines’: migrant workers’ conceptual framework of labour exploitation for health research and policy

**DOI:** 10.1136/bmjgh-2023-013521

**Published:** 2024-02-05

**Authors:** Sabah Boufkhed, Nicki Thorogood, Cono Ariti, Mary Alison Durand

**Affiliations:** 1 Department of Health Services Research and Policy, Faculty of Public Health and Policy, London School of Hygiene & Tropical Medicine, London, UK; 2 Humanitarian and Conflict Response Institute, The University of Manchester, Manchester, UK; 3 Department of Public Health, Environments and Society, Faculty of Public Health and Policy, London School of Hygiene & Tropical Medicine, London, UK; 4 Centre for Trials Research, Cardiff University School of Medicine, Cardiff, UK; 5 Department of Medical Statistics, Faculty of Epidemiology and Population Health, London School of Hygiene & Tropical Medicine, London, UK

**Keywords:** Health policies and all other topics, Health policy, Public Health, Other study design

## Abstract

**Background:**

The exploitation of migrant workers ranks high on global political agendas including the Sustainable Development Goals. Research on exploited workers, using assessment tools where exploitation is defined by professional experts, indicates serious health concerns and needs. Yet, migrant workers are rarely asked about their understanding of a phenomenon they may experience. Our study aimed to conceptualise ‘labour exploitation’ from the perspective of migrant workers employed in manual low-skilled jobs.

**Methods:**

Twenty-seven Latin Americans working in London (UK) participated in Group Concept Mapping; a participatory mixed-method where qualitative data are collected to define a concept’s content and then analysed using quantitative methods to generate a structured conceptual framework. Participants generated statements describing the concept content during brainstorming sessions, and structured them during sorting-rating exercises. Multi-Dimensional Scaling and Cluster Analysis were performed, generating a conceptual framework that clarified the dimensions, subdimensions and constituent statements of the concept of labour exploitation from migrant workers’ perspectives.

**Results:**

Three key dimensions were identified: ‘poor employment conditions and lack of protection’, covering contractual arrangements and employment relations; ‘disposability and abuse of power’ (or ‘dehumanisation’) covering mechanisms or means which make migrant workers feel disposable and abused; and ‘health and safety and psychosocial hazards’ encompassing issues from physical and psychosocial hazards to a lack of health and social protection. ‘Dehumanisation’ has not been included in mainstream tools assessing exploitation, despite its importance for study participants who also described harsh situations at work including sexual, physical and verbal abuse.

**Conclusion:**

Our study provides a conceptual framework of labour exploitation that gives voice to migrant workers and can be operationalised into a measure of migrant labour exploitation. It also calls for the dimension ‘dehumanisation’ and structural forms of coercion to be integrated into mainstream conceptualisations, and their workplace hazards to be urgently addressed.

**WHAT IS ALREADY KNOWN ON THIS TOPIC**

- Migrant worker exploitation is increasingly studied in health research, and findings indicate serious health concerns.
- Tools to assess exploitation use expert definitions of exploitation and overlook views of migrant workers exposed to labour exploitation, which may hinder the identification of important dimensions of exploitation affecting their health.

**WHAT THIS STUDY ADDS**

- Latin American migrant workers in manual low-skilled jobs in London participated in a mixed-method study and identified three key dimensions of labour exploitation: ‘poor employment conditions and lack of protection’; ‘health and safety and psychosocial hazards’ and ‘disposability and abuse of power’ which corresponds to ‘dehumanisation’ and is lacking in mainstream definitions.

**HOW THIS STUDY MIGHT AFFECT RESEARCH, PRACTICE OR POLICY**

- The conceptual framework highlighted three key dimensions, and their components, that matter to migrant workers, and could be operationalised as a measure of exploitation for health research and policy.
- Our findings call for including the ‘dehumanisation’ dimension and structural forms of coercion in mainstream tools assessing exploitation and its impact on migrant health, and for urgent research on and addressing of sexual, physical and verbal abuse experienced by migrants working in low-paid manual jobs.

## Introduction

The exploitation of migrant workers ranks high on global political agendas, such as the Sustainable Development Goals (SDGs)^1^ and Global Compact for Migration.^2^ The term ‘labour exploitation’ is often used by researchers, politicians or the media to refer to a wide range of issues ranging from precarious labour conditions through poor salaries to situations of modern slavery.^3–6^ In the UK, exploitation is discussed in both the Modern Slavery and Immigration Acts, thereby connecting exploitation and migration.^7–9^

However, the dearth of research on migrant workers’ exploitation and health has been highlighted in the past few years,^10 11^ and the lack of a standard conceptualisation has hindered the development of quantitative assessment of labour exploitation’s impacts on migrant workers’ health. Issues of labour exploitation in the health field have been mostly explored conceptually and in practice through two schools of thought.^12 13^ The Human Rights School focuses on severe and criminal forms of migrant worker exploitation such as modern slavery and human trafficking, and emphasises exposure to violence and physical harms.^14^ The Social Determinants of Health School focuses on structural aspects of exploitation such as precarious and other employment and working conditions, and emphasises mental health concerns such as stress, depression and anxiety.^15^

Boufkhed *et al* proposed a standardisable conceptual framework of migrant worker exploitation in low-paid jobs for health, bringing together the two schools of thought based on professional experts’ perspectives.^13^ They argue that their framework could be culturally and contextually adapted. Existing labour exploitation conceptualisations have ignored what migrant workers themselves consider exploitative, which hinders a full understanding of the concept and its health impacts. Our study addresses this gap in understanding.

The International Labour Organization defines migrant workers as ‘*international migrants who are currently employed or are unemployed and seeking employment in their present country of residence’*.^16^ There are 169 million migrant workers worldwide, with the majority located in high-income countries.^17^ Migrant workers are mostly employed in low-skilled jobs deemed exploitative.^18 19^ These are manual jobs generally requiring no or few skills to enter the position.^20^ The UK Office for National Statistics defines them as jobs requiring competence achieved when completing compulsory education and some work-related training.^21 22^ This includes jobs such as cleaners, catering assistant or porters. Migrants are known to face increased vulnerability, including the risk of human trafficking and other violations of their human rights.^14 18 23^ In this paper, the term migrant worker refers to international migrants who are currently employed in a manual low-skilled job in their present country of residence, which in our research is the UK.

There are almost six million migrant workers in the UK.^24^ Ongoing debates on migration and political claims of a will to ‘*create a hostile environment’* for migrants,^4 25 26^ and discussions surrounding the Immigration Bill and Brexit^27 28^ have created a political context placing migrant workers at increased risk of exploitation, especially those employed in manual low-skilled jobs.^29–31^ Meanwhile, the government has attempted to lead global fights against modern slavery and labour exploitation.^4^ The UK was the first country with a law designed to explicitly fight ‘*modern slavery’* (ie, Modern Slavery Act).^7^ In parallel, it has created a Directorate of Labour Market Enforcement overseeing the fight against exploitation in the UK.^32^ This directorate is framed within the 2016 Immigration Act and focused on migrant workers’ exploitation,^8^ making issues of exploitation and immigration intertwined.

Our study focuses on Latin American (LA) migrants working in manual low-skilled jobs in London (LAW). LAs in the UK are a heterogeneous group and the second fastest-growing immigration group.^33 34^ This population displays characteristics that make them prone to labour exploitation and facilitates their identification as a ‘population’. In addition to common vulnerabilities shared with other migrants such as language and limited rights access, they were identified as an ‘invisible’ migrant group facing the risk of going unrecognised as victims of criminal exploitation,^35 36^ and researchers and the media have documented their employment conditions as ‘exploitative’.^34 37–39^ They have self-organised to be identified as a ‘LA’ community and have been vocal in fighting against migrant workers’ exploitation in low-paid sectors.^36^ This has also allowed for safer engagement compared with other groups of migrants while avoiding unintentional harm when seeking their opinions on a sensitive topic such as exploitation. This population is mostly based in London and affected by different vulnerabilities to labour exploitation.^34 36^ Many LAWs have obtained a European passport that grants them the right to remain and work in the UK, though some have an irregular status. A high proportion work in manual low-skilled jobs, such as cleaning or hospitality. Specific difficulties in identifying human trafficking within this community due to cultural and employment specificities have also been acknowledged.^40^

Our study aims to conceptualise ‘labour exploitation’ from the perspective of migrant workers employed in manual low-skilled jobs, by identifying its dimensions, subdimensions and constituent items. It offers new insights into how labour exploitation is experienced or perceived by a group rarely engaged with the design of a research or assessment tool.

## Methods

### Patient and public involvement

Our research methodology is participatory by nature, involving low-paid migrant workers in the generation of a conceptual framework of labour exploitation as experienced by this group. As detailed below, in preparation for the main components of the research, key informant interviews were undertaken with representatives of organisations supporting migrant workers and with migrant workers themselves in part to ensure that our research design and recruitment methods were tailored appropriately to this population. We disseminated the preliminary findings for feedback from the population during a public engagement event in September 2019 in London.

### Theoretical framework and target population engagement

This study is framed within a psychometric and social epidemiology approach, and employed a mixed-methods methodology within a pragmatic epistemology.^41 42^ It is part of a wider research project (SB’s doctoral thesis^43^) that aimed ‘to clarify the concept of labour exploitation focusing on migrants working in manual low-skilled jobs, by providing a structured conceptual framework using professional experts’ and migrant workers’ voices (and building on) the growing conceptualisation of labour exploitation as a continuum ‘between decent work and forced labour’.^44^ It aimed to develop a conceptual framework for migrant labour exploitation by collecting data from various stakeholders across different fields, disciplines and experiences and to lay the ground for developing a measure of it, in order to foster quantitative research aiming at assessing its impact on health.

To ensure measurement validity when developing a measure, Streiner and Norman’s measurement steps^45^ and Cwikel’s SOCEPID framework for social epidemiology^46^ emphasise the need to clearly define the concept we intend to measure, and to include the target population in its definition for cultural and contextual adaptation. In this study, we engaged migrant workers in the preparation and data collection for the conceptualisation of exploitation. It complements an initial study in which professional experts developed a standardisable ‘skeleton framework’ of migrant labour exploitation adaptable for different populations and contexts^13^ to assess its impact on migrants’ health.

A conceptual framework is a ‘*system of concepts, assumptions, expectations, beliefs, and theories that supports and informs [the] research’*.^47^ One of its key functions is to facilitate the generation of knowledge in a structured way, including as ‘concept maps, mind maps or conceptual diagrams’.^48^ Novak and Cañas described concept maps as ‘*graphical tools for organising and representing knowledge’*.^49^ Group concept mapping (GCM) creates a common concept map using statistical analysis of individual contributions. The resultant framework is easily operationalisable and GCM is increasingly used as the first part of measurement tools development.^50 51^

### Design

GCM^51 52^ was considered the most suitable method for addressing the broader research aims and provide the basis for developing a potential measure of migrant labour exploitation. This is a participatory mixed-method approach that leads to the clarification of the content of an abstract and complex concept and has been used to address the first step of measurement development (ie, concept definition).^50 51 53 54^ Its design can be described as a sequential-dependant mixed-method design of equal status (QUAL→QUANT→qual) for the purposes of exploration and instrument development (framework or measure).^55–57^ Qualitative data are initially collected to capture and structure the concept content (statements) and then analysed using quantitative (multivariate) analysis to generate concept maps. On two-dimensional (2D) concept maps, each point represents an item (in this study, a statement describing a situation considered exploitation) and each cluster a dimension of the concept. The qualitative findings are also used to refine and illustrate the quantitative findings.

GCM has six phases: (1) ‘preparation’ (including sampling and preparation for the data collection); (2) ‘statement generation’ through brainstorming (ie, what constitutes migrant labour exploitation); (3) ‘sorting-rating’ to structure the brainstormed statements; (4) ‘multivariate analysis’ (Multi-Dimensional Scaling and Cluster Analysis) to produce concept maps; and (5) and (6) the ‘interpretation’ and ‘utilisation’ phases, respectively.

### Recruitment strategy and key informant interviews

**Figure 1:**
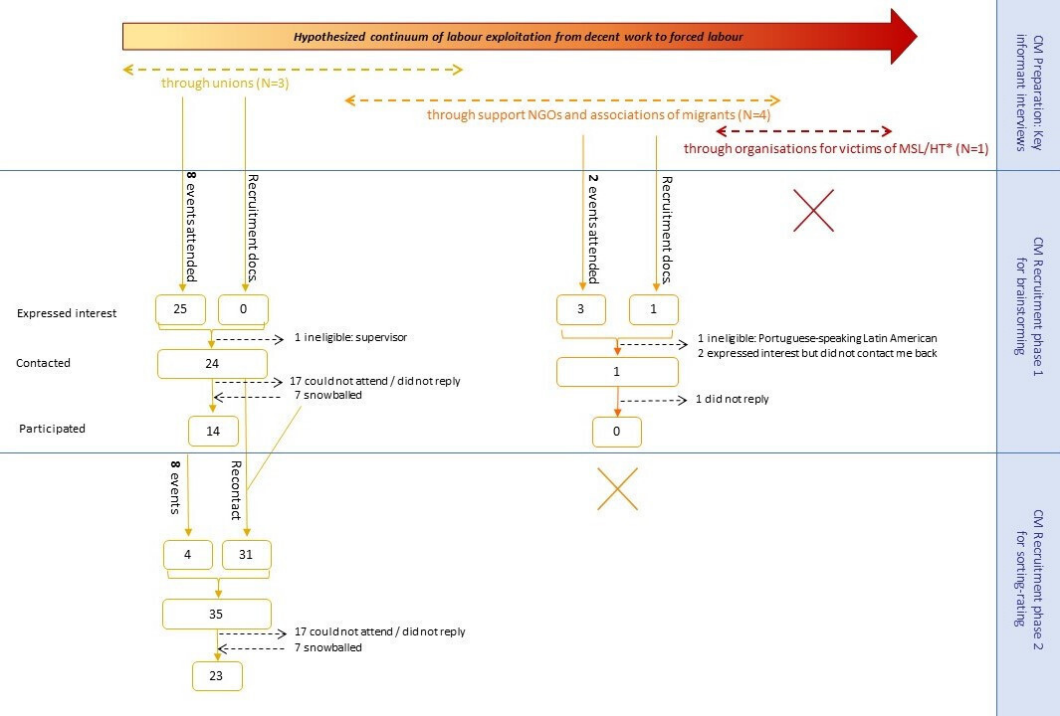
Participant flow describing the recruitment outcome per recruitment path. CM, Concept Mapping; NGOs, Non-governmental Organisations; MSL, Modern Slavery; HT, Human Trafficking.

The GCM target population was LAWs who may have faced situations of labour exploitation. Recruitment, as initially planned, was to be through three types of organisations supporting workers along a continuum of exploitation (from the less severe to the extreme exploitation) and through snowballing (see figure 1):

- Unions: This path was expected to capture experiences on the ‘lower’ and ‘middle’ part of the continuum, as their members may be more aware of their rights and entitlements, would be more likely to be documented and have access to support.
- Associations supporting (LA) migrants: To capture experiences on the ‘moderate’ to ‘extreme’ part. Their members might be in more vulnerable situations than those who were union members, and some LA associations have reported supporting victims of exploitation, including possible human trafficking.^40^
- Organisations supporting modern slavery victims: To cover the ‘extreme’ part. (As explained below this route was removed following a key informant’s advice).

As GCM had not previously been used with a potentially vulnerable population to generate the content of a sensitive concept such as ‘exploitation’, the preparation phase was adapted to include key informant interviews to build rapport with the community, ensure participants’ safety and comfort, and assess the recruitment and sampling strategies. Key informants were leaders of the organisation types listed above who were familiar with LAW experiences and worked with potentially exploited workers (N=3 union leaders; N=5 leaders of organisations supporting LAs/migrants; N=1 leader of a modern slavery/human trafficking organisation), as well as LAWs meeting the inclusion criteria (see below) and speaking English (N=3). We recruited key informants by contacting organisations which may support LA workers in London by email and during public events in which they participated.

These interviews aimed to: (1) undertake a preliminary exploration of the concept of migrant labour exploitation in the UK in preparation for the GCM and (2) seek advice on the practical aspects of arranging the brainstorming exercise with LAWs (eg, recruitment opportunities, preferred locations, potential support available to participants, translation needs, sensitive or taboo topics). Importantly, the modern slavery organisation leader confirmed that we should exclude modern slavery victims to avoid retraumatising individuals, as suggested in guidelines.^58^ The interviews with LAW were designed to explore the concept initially with English-speaking members of the community. Their contributions also described how they conceptualised labour exploitation and were added to the brainstorming data. Findings of the qualitative interviews with organisation leaders are not reported here.

### Population, sampling and recruitment

The GCM sample consisted of immigrants aged 18 and older, born in a Spanish-speaking Latin American country, and working in a manual low-skilled jobs in London for at least 6 months. Portuguese-speaking workers were excluded because of language and resource constraints. Individuals known to have an irregular immigration status were also excluded, as were full-time workplace supervisors. The later were excluded because key informants suggested that they are often seen as exploiters and/or perpetrators of abuse.

Purposive sampling was used to reach the recommended GCM sample size of 10–40.^52^ We planned to recruit at least 20 participants: 10 LAWs through each of the first 2 routes above, with at least 2 groups of approximately 5 participants: 1 for men and 1 for women. This group size was expected to facilitate an engaging discussion while developing a variety of statements describing exploitation.

A Spanish-speaking research assistant (RA) was recruited, trained and supported recruitment and data collection. GCM participants were recruited at public events attended by LAs and through snowballing. All participants who were contacted for and/or participated in the brainstorming were invited to participate in the sorting-rating phase along with new participants recruited throughout the data collection phase. Recruitment outcomes are presented in figure 1.

### Data collection and management

#### Statement generation

A detailed brainstorming session guide and kit, and a demographics form were developed (SB),^43 59^ and the GCM brainstorming pilot tested. Face-to-face group or individual brainstorming sessions during which LAW generated statements describing migrant labour exploitation were conducted in Spanish in London (UK) in February and March 2017. Participants were asked to generate as many short statements as they wanted to complete the prompt ‘*Un trabajador migrante es explotado cuando…’* (*‘A migrant worker is exploited when…’*). The sessions were audiorecorded and transcribed to extract statements that may have been missed during the session.

During group sessions, statements generated were written on a paperboard and/or post-it notes. During individual brainstorming sessions, the generated statements were written in a large notebook so that the participants could see them as they talked. The first individual session was conducted in English at the participant’s request. Notes were also taken of statements generated throughout the sessions.

#### Data entry, reduction and synthesis to generate the final list of statements

**Figure 2:**
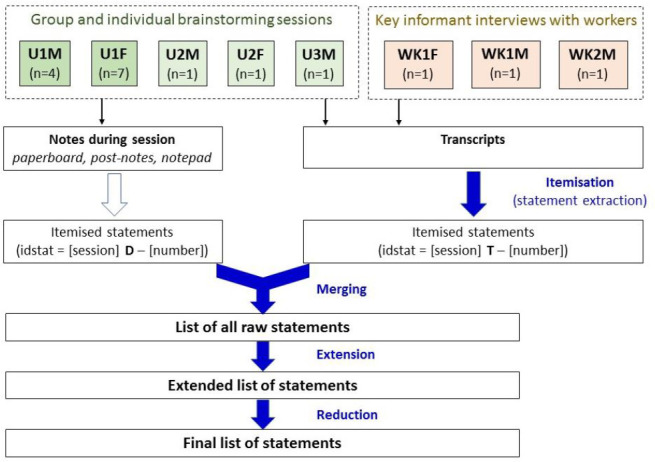
Data processing steps of the statements generation phase to produce the final list of statements.

Figure 2 depicts the process used to generate the final list of statements describing the concept content, and the session identifiers linked to the sources of the quotes included in the Results section.

A list of raw statements was generated by extracting them from the post-it notes, notebooks, paperboard, transcripts of brainstorming sessions and key informant interviews with LAW. The notes and transcripts were exported to an Excel spreadsheet where SB performed a statement extraction, that is, ‘itemisation’.^60^ Each statement was given an identification code (ID) to track its source (e.g., notebook, transcript). All statements extracted were merged to create a list of all the raw statements. This file contained ID statements (i.e., number corresponding to the statement), the statement in Spanish or English (depending on the session) and an English translation where needed.

This list was then reduced and synthesised until a final list of less than 100 statements was reached, as recommended by GCM developers.^52^ Following the example of Boufkhed *et al*,^13^ duplicates and statements deemed to be outside the study scope were deleted. Given the high number of raw statements, the statements were reduced further by combining those that were very specific or comparable experiences to create a more general, encompassing statement.^61^ The final list of 94 statements was translated into Spanish and back-translated by native Spanish-speakers.

#### Statements structuring

Following pilot testing, the sorting-rating tasks were performed individually during group or individual sessions. Participants structured the 94 statements that were displayed on cards (one statement per card) by organising them into piles ‘in a way that makes sense for you’,^52^ and named each pile. They then rated the importance of each statement in characterising a situation as exploitative, using the list of the 94 randomly ordered statements and a 5-point Likert scale (1 ‘relatively unimportant’ to 5 ‘extremely important’). For each participant, an Excel file was created with the outcomes of the sorting on one sheet and the ratings on another. Data were then imported and verified into Stata V.14.^62^

### Data analysis

Descriptive statistics were used to describe participants’ characteristics. The sorting and rating results were quantitatively analysed^63^ using SPSS V.24 (IBM SPSS software (www.spss.com)). The full details of the analysis are described elsewhere^13^ and summarised here. First, the sorting results were analysed using Multi-Dimensional Scaling (MDS) which generated a 2-D map. The closer the points on the 2-D map the more conceptually similar the corresponding statements are.

**Figure 3:**
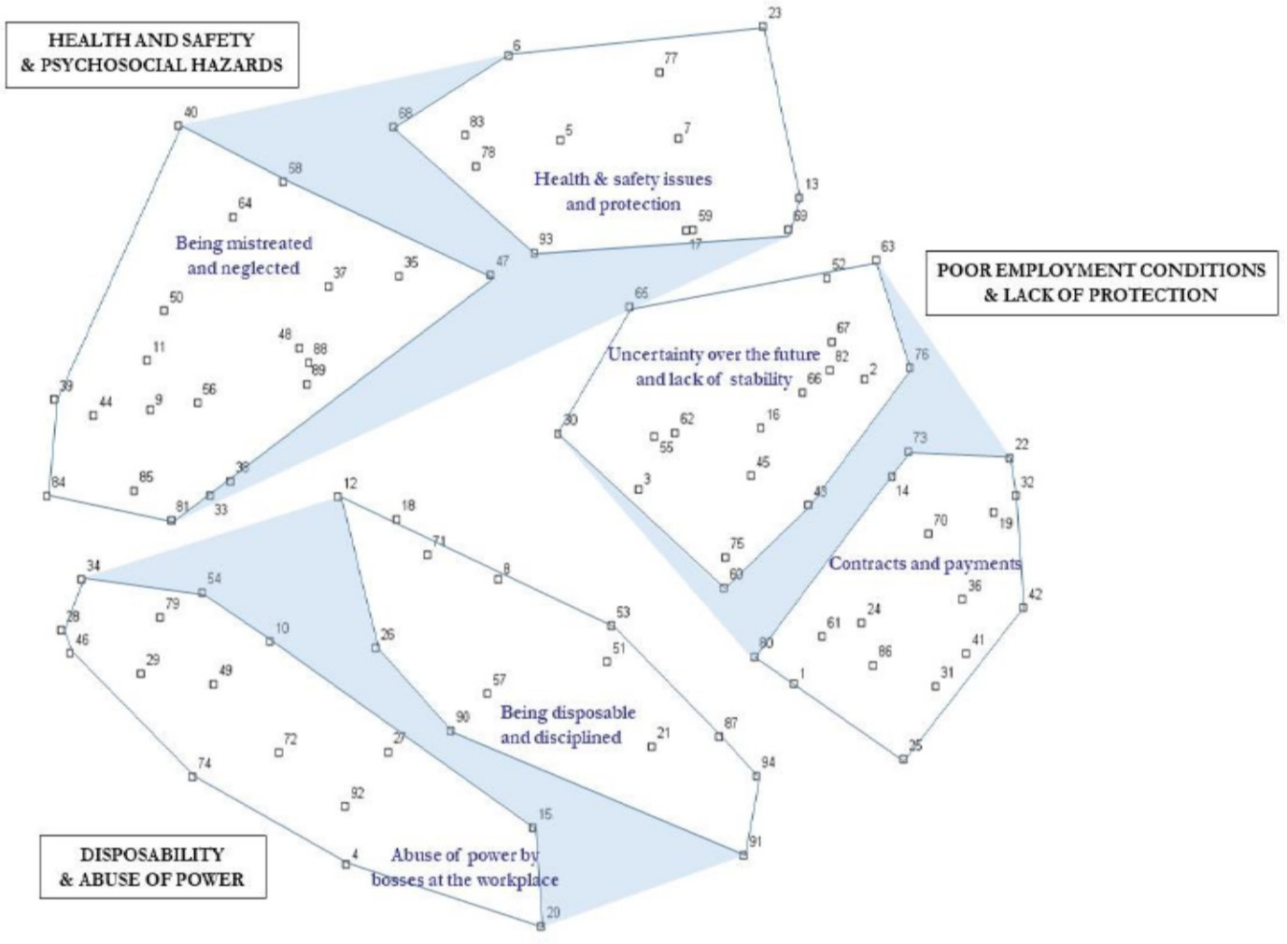
Structured conceptual framework of migrants’ labour exploitation tailored for and by Latin American migrants working in manual low-skilled jobs in London.

Second, the MDS outputs (points coordinates) were analysed using a hierarchical cluster analysis (CA), which helped delineate clusters corresponding to the concept dimensions. The clusters were named using some of the participants’ labels. Finally, clusters that were conceptually similar were regrouped into ‘regions of meaning’^63^ which represented key dimensions. The final conceptual framework displayed points corresponding to statements (or items), clusters corresponding to subdimensions and shaded regions of meaning corresponding to the key dimensions (figure 3).

## Results

### Participants

Participants’ characteristics are presented in table 1. The sample consisted of 27 LAW, with similar proportions of men and women. On average, they were aged 45 years (SD=10.9), had lived in the UK for 9 years (SD=8.5) and worked in London for 7 years (SD=5.9). Most participants reported some deficits in their English language skills, especially in speaking. They were almost all cleaners (N=22/27), mainly employed by outsourcing companies (63%), and about half were working part time (48%).

**Table 1:** Characteristics of participants in the Group Concept Mapping with Latin Americans migrants working in manual low-skilled jobs (N=27)

Participants’ characteristics	Overall (n=27)	
	n	%
Female	**11**	**40.7**
Country of birth		
Colombia	15	55.6
Ecuador	7	25.9
Other*	5	18.5
Level of English		
Fluent or almost	6	22.2
Can speak but cannot read/write	2	7.4
Can read/write but cannot speak	6	22.2
Speak, read/write with difficulty	11	40.7
Cannot speak, read/write	1	3.7
Missing	1	3.7
Way she/he found the current job:		
Someone she/he knows told him/her about the job	21	77.8
Found it him/herself	3	11.1
Other (unemployed)	1	3.7
Missing	2	7.4
Type of employer		
Employed by the workplace where she/he works (in-house/internal employee)	6	22.2
Employed by an outsourcing company	17	63.0
Unemployed	1	3.7
Other†	2	7.4
Missing	1	3.7
Current job title		
Cleaner	22	81.5
Ex-cleaner	1	3.7
Gardener	1	3.7
Bartender	1	3.7
Cook	1	3.7
Interpreter	1	3.7
Highest level of education completed		
Primary school	1	3.7
Secondary school/A-levels	13	48.2
Higher education	7	25.9
Vocational training	2	7.4
English certificate	1	3.7
Missing	3	11.1
Working full time		
Full time	13	48.2
Part time	13	48.2
Unemployed	1	3.7

*Spanish-speaking country of central and South America.

†Includes: ‘both in-house and outsourced’ and ‘retired’.

The sociodemographic distributions remained similar for both steps in the GCM exercise, although there were slightly more men in the sorting-rating phase (60%) (see online supplemental additional file 1 for details). Overall, there was no major difference in the distribution of women and men’s characteristics, except that a higher proportion of men were in a full-time position (respectively, 63% vs 27%), directly employed by their employer (in-house) (31% vs 9% for women) and had achieved higher education (31% vs 18%).

10.1136/bmjgh-2023-013521.supp1 Supplementary data

### The conceptual framework and migrant workers’ conceptualisation

The structured conceptual framework of labour exploitation from the perspective of LAWs is presented in figure 3. Table 2 details the concept content: statements, their rating and an ID corresponding to the point number on figure 3. The map reveals three key dimensions (shaded in blue on the figure): (1) ‘poor employment conditions and lack of protection’; (2) ‘disposability and abuse of power’ and (3) ‘health and safety and psychosocial hazards’.

**Table 2:** Cluster content and importance ratings of the concept mapping with Latin Americans working in manual low-skilled jobs in London

ID	Dimensions, subdimensions and statement label	Mean	SD
Poor employment conditions and lack of protection			
Uncertainty over the future and lack of stability		4.41	0.23
60	She/he is not paid the right amount of hours at the end of the month	4.87	0.34
52	She/he has to complain to get his/her payment or holidays entitlements owed	4.78	0.52
30	She/he can be fired without justification	4.73	0.70
76	She/he is not given the opportunity to read and understand the contract	4.61	0.66
62	She/he is not paid at the end of the month	4.55	0.74
65	She/he is fired when coming back from authorised absence or holidays	4.39	0.94
75	She/he is constantly asked to wait for his/her contract to be updated	4.39	0.78
63	She/he does not have paid holidays	4.36	0.85
45	His/her documents are used to hire another worker	4.35	1.19
2	She/he is not given a contract	4.35	1.11
16	She/he does not receive training explaining what and how to do his/her job	4.35	0.98
67	She/he does not know how or to whom to complain to about a problem at work	4.30	0.88
3	She/he is told that she/he will receive no training or protective equipment because she/he works fewer hours than the other workers	4.26	1.05
55	She/he is obliged to take fragmented/scattered holidays	4.26	0.81
82	His/her working hours are fragmented	4.22	0.85
66	She//he is not paid his/her full lunch break	4.09	1.08
43	His/her working hours are in different part of town	4.04	1.30
Poor contract and payment issues		4.40	0.20
70	She/he is not paid for extra hours/work	4.78	0.42
36	She/he is given a part-time contract while she/he actually works full time	4.64	1.00
19	His/her holiday entitlement is lower than what she/he should have for the number of hours actually worked	4.59	0.73
73	She/he is paid less than the living wage	4.57	0.95
1	She/he is outsourced	4.52	0.67
25	She/he is paid less than the minimum wage	4.50	1.06
14	She/he has no pay rise after working many years for the same company	4.43	1.04
80	She/he works at night for the same salary as during daytime	4.43	1.04
41	She/he is not given detailed information about the contract	4.39	0.72
42	His/her holidays payment is given to someone else	4.36	1.09
22	She/he does not have the same pension benefits than the in-house workers	4.35	0.71
86	She/he has a zero-hour contract	4.30	1.15
31	She/he has no legal documents	4.23	1.23
24	She/he is fired because she/he had an older contract with better conditions	4.18	1.10
61	She/he does not receive payslip	4.13	1.14
32	She/he has a short-hour contract	4.05	1.05
Disposability and abuse of power (or dehumanisation)			
Being disposable and disciplined		4.52	0.36
90	She/he is forced to do a physical task that should be done by two persons	4.91	0.29
53	His/her quantity of work increases without pay raise	4.78	0.42
57	She/he is forced to work more for the same salary to keep his/her job	4.74	0.62
91	She/he has a heavier workload than his/her colleagues who were recruited recently	4.73	0.46
12	She/he has to cover without payment another person’s absence	4.70	0.56
87	She/he is paid less than another worker doing the same job in the same company	4.70	0.56
71	She/he is given a disciplinary sanction if she/he cannot finish his/her work within allocated time	4.65	0.57
18	She/he is pressured to do more work than feasible in the allocated time	4.63	0.71
26	She/he is taken to a disciplinary/investigation meeting for complaining	4.48	0.59
94	She/he lacks materials to work	4.39	0.72
51	She/he is told on the day not to come because there is no work	4.36	1.00
8	She/he is afraid to lose his/her job if she/he joins a union	4.13	1.36
21	She/he is given a couple of hours work in the middle of the night	3.52	1.17
Abuse of power by bosses at the workplace		4.48	0.25
54	She/he is not treated as a human being	4.87	0.34
49	She/he is discriminated against at work	4.78	0.52
79	She/he is threatened with being sacked if she/he cannot perform his/her job tasks due to an injury	4.78	0.52
74	His/her boss abuse his/her position to date him/her	4.70	0.93
10	She/he is threatened with being sacked if she/he goes on strike	4.57	0.84
15	His/her boss refuses to pay him/her all the hours worked	4.57	0.73
27	She/he is given more workload if she/he complains	4.52	0.79
29	She/he is bullied	4.52	0.73
92	His/her boss is always supported when there is an investigation on him/her	4.48	0.79
72	His/her boss asks him/her money because she/he covered him/her when she/he was absent	4.43	0.99
46	His/her boss tries to touch / touches him/her	4.36	1.26
28	His/her boss tries to fire him/her because she/he refused a date	4.27	1.03
20	His/her boss is not trained to do his/her job and manage workers	4.13	1.06
34	His/her boss shows favouritism in work allocation	4.13	0.92
4	She/he cannot work peacefully because the boss constantly changes his/her tasks or working area	4.05	1.09
Health and safety and psychosocial hazards			
Mistreated and neglected		4.45	0.26
89	She/he is not offered solutions to issues at work but told to leave if not happy	4.83	0.39
88	She/he is humiliated at work	4.77	0.43
44	His/her boss creates a hostile environment to force him/her to quit	4.70	0.56
48	She/he is physically assaulted	4.65	0.88
84	She/he is yelled at by the boss	4.65	0.65
11	She/he is psychologically abused	4.65	0.57
40	His/her boss refuses to adapt his/her duty if She/he is injured or pregnant	4.59	0.96
64	His/her boss’s bad communication prevents his/her issues to be acknowledged	4.57	0.59
37	She/he has no right to eat and is not given water at work	4.55	0.74
39	She/he is insulted by his/her boss	4.52	0.79
58	She/he is threatened with being sacked when she/he cannot work because She/he is sick	4.52	0.67
38	She/he is threatened of disciplinary sanctions	4.43	0.73
56	His/her work is never well-done in the eyes of the supervisor	4.39	0.66
33	She/he cannot complain as she/he fears losing his/her job	4.35	0.93
85	She/he is threatened with being sacked if she/he wants to complain	4.30	1.06
35	She/he cannot speak the language	4.26	1.05
9	His/her bosses do not let him/her rest	4.17	0.89
81	She/he is scared of his/her boss	4.05	1.40
47	She/he is not given free time for his/her own activities	4.04	1.33
50	She/he is forbidden to have kids	3.91	1.51
Health and safety issues and lack of health protection		4.53	0.14
5	She/he is not paid by sick pay from the first day of sickness (with medical justification)	4.74	0.75
77	She/he does not have sick pay	4.70	0.63
6	She/he is not covered/compensated in case of a work accident	4.65	0.78
7	She/he is not informed about workers’ rights	4.65	0.65
83	She/he gets sacked following a work injury/accident	4.61	0.94
59	She/he is tricked into signing a document telling She/he received health and safety training…	4.57	0.66
93	She/he is told she/he is not entitled to sick pay because She/he works part-time	4.52	0.79
68	She/he gets injured because she/he had to rush to do his/her work	4.48	0.85
23	She/he does not receive health and safety training	4.45	1.01
78	She/he has no right to leave work to care for his/her family	4.41	1.05
69	She/he loses money when She/he is sick	4.39	1.03
13	She/he can only afford to live in a shared overcrowded house	4.36	0.85
17	She/he does not receive the adequate protection equipment	4.30	1.02

The GCM approach involves identifying regions of meaning. These are regions on the maps that bring together adjacent clusters that share some underlying commonality. It became apparent that adjacent clusters which are within the regions of meaning (in blue on the figure 3) were actually describing situations that could be seen as happening in different contexts or settings:

- ‘Poor employment conditions and (social) protection’ are situations involving macrolevel context (ie, organisations’ and countries’ regulations). Hence, they are more ‘structural’ in nature.
- ‘Health and safety and psychosocial hazards’ encompass situations happening at the workplace and refer to supervisors or managers’ practices.
- ‘Disposability and abuse of power’ are situations which happen at an intermediary (meso) level and can be seen as ‘institutional’ forms of exploitation as they seem to be a combination of managers’ practices facilitated by the institution in a climate of impunity and what participants felt were a lack of ‘care’ or consideration for them.

The key and subdimensions are described below and illustrated by quotes from the sessions that generated statements to give voice to the workers.

#### Poor employment conditions and lack of protection

The key dimension ‘poor employment conditions and lack of protection’ includes the subdimensions ‘uncertainty over the future and lack of stability’ and ‘poor contract and payment issues’. It describes aspects of employment conditions and protections that are generally found in contractual arrangements and defines employment relations, as highlighted by a LAW:

When you do not have a contract or an explanation of which area you have to clean, day after day, they add things to you, and how could you say no.”(Individual brainstorming with woman—U2F)

Participants expected contractual arrangements to foster a sense of security and capacity to plan their futures. However, they viewed some contracts, such as zero-hour contracts, as exploitative, and experiences illustrated by this dimension reflect the lack of a ‘safety net’ when, for instance, they experienced irregular or delayed payment. This dimension implies some structural exploitation, and the following quote highlights the relation between the two clusters:

people with zero-hour [contracts] do not have the right to take paid holidays, they do not have paid holiday […] You can take your holidays, but they do not pay you […] there is no job stability for those people, which is crucial! […] All contracts have to be under this regime: all with paid vacations, all with sick pay and all… with job stability! (Individual brainstorming with man—U3M)

The subdimension ‘uncertainty over the future and lack of stability’ covers statements indicating employment conditions preventing migrant workers from planning their short-term future (eg, #60 ‘*not paid the right amount of hours at the end of the month’*, or #82 ‘*working hours are fragmented’*) or longer-term (eg, #30 *‘can be fired without justification’*). Statements like #62 ‘*not paid at the end of the month’* or #65 ‘*fired when coming back from authorised absence or holidays’* also illustrate a sense of unpredictability.

‘Poor contract and payment issues’ refers to specific contractual arrangements—like zero-hour contracts or outsourcing (respectively statements #86 and #1)—seen as exploitative, and problems with payment like *‘being paid less than the minimum wage’* (#25). This subdimension also covers migration issues like having ‘*no legal documents’* (#31). Statements like 14 ‘*[having] no pay rise after working many years for the same company’* (#14) or ‘*working at night for the same salary as during daytime’* (#80) may reflect migration experiences as most have previously worked in other countries with more labour rights.

#### Disposability and abuse of power

This dimension is composed of ‘being disposable and disciplined’ and ‘abuse of power by bosses at the workplace’, and refers to mechanisms or means which make migrant workers feel disposable and abused.

They threaten people […] They put them one… there is this [complaint] form […] it’ s a paper that tells you […] I'm going to pass this complaint because you did not listen to me to do the work, you have to, they force you to sign it. […] At the third of these papers, they can suspend or sack you from the company. (Individual brainstorming with man—U3M)

These subdimensions were similar conceptually and illustrate that workers feel disposable when bosses or companies demand tasks be done whenever and in whatever way they want, without consideration for workers as individuals:

I have witnessed that a colleague was here with a swollen lump, with fever, and worked. I go, and I told the supervisor: why? You are a person just like her, why do you not send her home? [Why] do you permit those things? And, in all truth, instead of helping, they crush them more so that these people do not rise. (Group brainstorming with women—U1F)

The statement which was most representative of LAW’s feeling of exploitation was ‘*not treated as a human being’* (statement #54 in ‘Abuse of power’). It was repeatedly used in sessions with LAW and underscores their feeling of being treated as commodities instead of humans:

those at the top [have] to be aware, that those who work are human beings, that we work with human beings, that we have limitations, and physical limitations too; and that not everyone work equally. […] In the end those who do the work for [them] to live well, are those at the bottom. Well then… take the time to visit people, to ask their opinion, see what programs they have. That is very important, know them, know the base, the workers, […] see how they work, learn their names, ask how they live, how long have they been working in the company, what problems they have, give the possibility for them to communicate with you. (Individual brainstorming with man—U3M)

The subdimension ‘Being disposable and disciplined’ describes how workers feel threatened and used as if they have no personal life when they are, for example, asked to ‘*work two hours in the middle of the night’* (#21) or ‘*told on the day not to come because there is no work’* (#51). Statements highlighted their perceived inability to complain about their conditions for fear of being disciplined or punished (eg, ‘*taken to a disciplinary/investigation meeting for complaining’* (#26) or ‘*given a disciplinary sanction if s/he cannot finish his/her work within allocated time’* (#71)). They perceived that this forced them to accept high workloads without compensation (#18 ‘*pressured to do more work than feasible in the allocated time’* or #57 ‘*forced to work more for the same salary to keep his/her job’*). A LAW shared how *companies* treat them like machines without any agency:

They treat us like machines sometimes. They don’t… don’t… feel or think NOTHING about you! The only thing is you c[o]me here to do it your job. ‘ I don’t care if you have family, you are sick, or anything.’ […] they treat me like I'm a table or like a chair or… Only to doing that… and, and nothing more. And I say… why? I am a person […] But… they… they don’t care. (Key informant interview with woman worker - WK1F)

The subdimension ‘Abuse of power at the workplace’ exemplifies situations where they feel bosses at the workplace use their position and power to abuse and diminish migrant workers (eg, #72 *‘asking money to the worker when covering for him/her’*, *#4 ‘cannot work peacefully because the boss constantly changes his/her tasks or working area’*). Issues of bullying, favouritism, discrimination or bosses’ impunity (eg, #*92 ‘his/her boss is always supported when there is an investigation on him/her’*) were included in this subdimension that could be seen as *mechanisms* of exploitation. A LAW from the men’s group brainstorming shared:

they [the workers] say, ‘I do not have the time to clean the dust on those edges. I only have time to clean the tables and the board, ok, I do not have time to clean the edges’. She [the supervisor ] says ‘You have to do it, You have to do it’ […] the cleaners have complained several times about her, they say she is very, what is the name, she likes […] to be the best but has problems of disrespect for them. A lack of respect because everything has to be forced […] (Individual brainstorming with man—U3M)

Issues of sexual harassment perpetrated by bosses at the workplace were conceptualised as part of ‘abuse of power’ when they might be expected to be in the ‘health and safety’ cluster within ‘physical assaults’. This may indicate a perception that these are a combination of bosses’ impunity and power over women migrant workers, as illustrated in the women’s group brainstorming:

B: […] they think they have authority.

A: It usually happens: I had a manager who invited me to go out. And because I was not interested in going out, so when he saw that I was not interested in going out, he looked for all the necessary means to fire me. (Group brainstorming with women—U1F)

#### Health and safety issues and psychosocial hazards

This dimension incorporates issues ranging from physical and psychosocial hazards through mistreatment of workers and/or neglect at work, to a lack of health and social protection:

U2—I suffer, I am suffering now about… lack of PPE.

Interviewer—What do you mean?

U2—Yeah. I am sick, I am still sick. I asked since September [that] they bring me a jacket because I take rubbish outside the building. Every time I have to go out, I felt sick in that time, I asked them, but they refused to give me… when the manager at [company X] heard me with a cough [all the] time, they [spoke] with managers, and they gave me one old jacket from another company last week. […] They gave me the old jacket last week and then I have otitis […] And… I cannot go to work. I asked to my supervisor, he says maybe you can’ t earn money these days because this company do not pay for sick pay. (Individual brainstorming with man—U2M)

The statements included illustrate issues that may directly (or in the short-term) affect workers’ safety, health and personal life. For example, one woman in the women’s group brainstorming shared:

he said we were donkeys [i.e. stupid], so he took the mop bucket and kicked it, and hit me on the leg. […] I was already suffering by the way he treated us: not letting you work in one place quietly, he changed you, you were here now not there anymore, in half an hour he comes and [says] I changed you. And the way was humiliating. And after that I got sick, and I was very bad psychologically. Just listening to him I started to cry, tears came out just listening to the man and that’s why they gave me 7 months of sick leave. […] I still suffer from that, from the psychological and physical harassment. (Group brainstorming with women—U1F)

The subdimension ‘being mistreated and neglected’ describes mistreatment faced by workers at the workplace, be it physical (#48 ‘*physically assaulted’*) or psychological (eg, #84 ‘*insulted’* or #39 ‘*yelled at’*); and situations of neglect or carelessness, such as being given tasks that are not adapted when workers are pregnant or injured (#40). This subdimension includes issues that were widely reported in the GCM sessions, including LAWs feeling abused because of their lack of English-speaking skills and being ‘*scared of the boss’* (#81). It seems to include both *causes* and *consequences* of the statements within this cluster. The statement #50 ‘*s/he is forbidden to have kids’* is also included in this cluster, and we believe that participants may have randomly allocated it or interpreted it differently because it raised many questions during the sessions. Some did not believe that this could happen, and some (especially men) stressed that they did not understand its meaning.

The subdimension ‘Health and safety issues and lack of health protection’ covers more traditional health and safety issues related to sickness and accidents, lack of protective equipment, and poor health benefits. Statements related to workers’ personal circumstances, such as needing care leave or poor housing conditions, were also in this cluster. We expected the statement #7 ‘*s/he is not informed about workers' rights’* to be under ‘employment conditions’ rather than this subdimension, though participants may have perceived their lack of knowledge about their rights to be a cause of these health and safety issues.

## Discussion

Our research generated a contextually and culturally specific conceptual framework of migrant labour exploitation that details and clarifies a complex concept from the perspectives of migrant workers themselves. It highlights what matters to workers in their perceptions and experiences of exploitation through three key dimensions: ‘poor employment conditions and lack of protection’; ‘disposability and abuse of power’ and ‘health and safety and psychosocial hazards’.

It provides empirical evidence of the need to consider national contexts and specific populations in identifying situations as exploitative. Our work demonstrates the feasibility and relevance of the GCM method in identifying dimensions of a complex concept with a population under-represented in health research. It offers a rare channel for migrant workers to shape a research and policy tool by defining a phenomenon they experience. The dimensions identified can complement the standardisable ‘expert skeleton’ framework^13^ to design a measure of migrant labour exploitation that would be adaptable to contextual and populations’ specificities.

### An overlooked dimension: ‘disposability and abuse of power’ or ‘dehumanisation’

The dimensions ‘poor employment conditions and protection’ and ‘health and safety’ echo what we found in the ‘expert skeleton’ framework with professional experts.^13^ However, by following recommendations to better engage communities in research,^64–66^ our research revealed the content of the dimension of ‘disposability and abuse of power’ which reflects a widespread view in qualitative research and activism that migrant workers are treated like machines and not as humans.^67^ Despite its importance for the workers in our study, it is absent from measures of exploitation such as the International Labour Organization experts-based indicators for labour trafficking^68 69^ or Muntaner *et al*’s theory-based measures of exploitation.^70 71^

Haslam’s integrative multidisciplinary review of the concept of dehumanisation^72^ offers insights from psychology that can connect our empirical findings to theory and help identify pathways to ill health. He distinguishes ‘animalistic’ and ‘mechanistic’ dehumanisation. While animalistic dehumanisation relates to violence and viewing others as ‘subhumans’ (Haslam; p.259),^72^ mechanistic dehumanisation is described as a ‘disregard’ for others who are portrayed as ‘non-humans’.(Haslam; p.259)^72^

Haslam suggests that animalistic dehumanisation ignores individuals’ ‘unique human’ attributes encompassing more ‘sophisticated’ emotions not identified in animals (eg, intelligence and culture). He argues that animalistic dehumanisation can take ‘milder’, ‘daily forms’ and may be accompanied by ‘degradation’ (Haslam; p.258)^72^ and ‘violence’. (Haslam; p.255)^72^ We suggest that this concurs with LAWs’ descriptions of mistreatment experienced as a result of individual bosses’ abuse of power. ‘Abuse of power by bosses’ could therefore be relabelled ‘animalistic dehumanisation’, reflecting how supervisors may treat migrant workers as ‘subhumans’ (Haslam; p.258)^72^ that are ‘disregarded’.

In contrast, LAWs’ perspectives on workers being treated like machines under ‘being disposable and disciplined’ could potentially be viewed as mechanistic dehumanisation. Haslam argues that it denies the essence of human nature and is a ‘denial of individual agency (that) represents them as interchangeable (fungible) and passive’. (Haslam; p.258)^72^ LAWs may be seen by company owners as ‘socially distant’ (Haslam; p.262)^72^ (non-humans) which is reflected in the concept of ‘disposability’ that emerged in our work.

We argue that the dimension ‘disposability and abuse of power’ could therefore be renamed ‘dehumanisation’. This dimension of labour exploitation has been relatively overlooked in previous conceptualisations of labour exploitation in health. Still, it aligns with notions of dignity and respect, or alienation, which are present in EMCONET’s fair employment concept.^73^ It is also reflected in research reporting migrant workers’ perceptions of not being treated like humans^67^ and in major (migrant) workers’ campaigns in the UK and beyond on dignity and respect.^74–76^ Despite its importance, to date, the dimension has not been defined within measures or indicators of exploitation.^67 77 78^ Our work highlights its importance for migrant workers and details its components, and calls for exploring the mainstreaming of ‘dehumanisation’ into health research, practice and policy. Further research is needed to explore whether this dimension could be considered a core component of migrant workers’ exploitation or as a dimension specific to some sectors or group of migrants.

### Sexual abuse at work

The location of sexual abuse within the ‘disposability and abuse of power’ dimension (or ‘dehumanisation‘) may indicate that ‘women’ in situation of labour exploitation might be considered as ‘subhumans’ compared with men, corresponding to Haslam’s ‘animalistic dehumanisation’. This aligns with other research drawing a parallel between dehumanisation and work-related gender issues and abuse of women at work.^79 80^

Research on extreme forms of labour exploitation like human trafficking has shown that women face sexual abuse and violence even when trafficked for labour and not sexual exploitation.^81–83^ Yet, there is limited research assessing the extent of sexual misconducts experienced in lower-skilled jobs by non-trafficked people, despite the existence of surveys on physical and psychological violence (aggression, harassment) in the workplace.^84 85^

Our study raises serious concerns about sexual violence in the workplace, which needs urgently to be researched further and addressed, especially for migrant workers in manual low-skilled jobs. Cases of rapes, attempted rapes or molestation were reported during interviews with key informant organisations, but also during some individual face-to-face sorting-rating exercises with men who disclosed stories of sexual harassment and assaults triggered by the card ‘*his/her boss tries to touch/touches him/her’*. One shared that women were often ‘forced to date’ to obtain or keep a job. Another participant reported that a supervisor almost raped a woman at his workplace but was stopped by a security agent doing his patrol. He added that the manager covered up for the incriminated supervisor, hence emphasising the point on impunity. The investigation was still ongoing at the time of our research.

### Structures and severe forms of exploitation

The content of ‘poor employment conditions and lack of protection’ echoes literature on precarious employment which emphasises the lack of rights and/or ability to exercise them.^86^ It reflects structural aspects of exploitation through lacking rights or experiencing employment conditions perceived as exploitative (eg, zero-hour contracts). These aspects may be specific to the UK labour market which is characterised by a flexible labour market where wages and job quality have decreased following the 2007/2008 financial crisis.^87^ It highlights that, despite being lawful, some employment conditions such as being outsourced, not given a contract or given a few hours of work in the middle of the night are perceived as exploitative by LAWs. This may also reflect participants’ previous experiences of migration and deskilling, which may be a characteristic of the LA population in the UK.^34^

Most of LAWs’ experiences concur with employment and working conditions seen in research on migrant workers in low-paid sectors.^67 88–90^ While our participants were mostly recruited through unions which we hypothesised would cover ‘lower’ levels of exploitation, the experiences described could be argued to match many of the UK modern slavery indicators.^91^ Therefore, either participants were exposed to higher levels of exploitation, or the under-regulation of the service sector and employment conditions in the UK are structurally exploitative, as suggested by Marxist views and a Social Determinant of Health approach to exploitation.^12 13^ Our findings further support Buller *et al*’s suggestion that situations of extreme labour exploitation seem to coexist with less severe cases,^92^ and the need to connect the Human Rights and Social Determinants of Health schools of thoughts.^13^ It is worth adding that migrant workers with a more precarious immigration status, or with less knowledge of support systems, may be facing worse conditions than those reported above.

### Strengths and limitations

This research provides the first conceptual framework of labour exploitation directly developed by a group of migrant workers in the UK. We used GCM, which is a robust methodology, to build an operationalisable conceptual framework, and proposed methodological improvements for using GCM with underrepresented populations. To the best of our knowledge, it is the first time that the GCM method has been used with migrant workers. The identification of dimensions and their content adapted to a migrant population in the UK is particularly timely given the UK’s political context and in terms of supporting the 2030 SDGs on Decent work and fight against exploitation. Our sample has a size within the range of other CMs in the existing literature,^54 93^ and its composition is consistent with the distribution of Spanish-speaking LAs in London.^36^

The generalisability of our study is limited by the non-random sampling used and a sample size which may be considered relatively small, although it compares favourably with other GCM studies. Donnelly^93^ systematically reviewed doctoral dissertations using GCM and found a mean (SD) of 49 (78.16) participants in the brainstorming phase; 12.33 (3.61) participants with completed the sorting exercise and 35 (30.84) the rating exercise. In Rosas and Kane 54 which conducted a pool analysis of 69 GCM that used a commercial software for GCM, there were 24.62 (15.30) sorters and 81.77 (69.83) raters. Limited access to and vulnerabilities of the population led to a sample composed of Spanish-speaking LAs and union members and people connected to them. The sample composition and size may limit the generalisability of the findings, including by underestimating the potential situations considered as exploitative. This sample was, however, relevant for the study purposes, respected ethical considerations, and echoed situations described as exploitative in the literature. Existing research conducted in London with LAs and other migrants in similar work sectors suggests that these views may be generalisable.^34 36 67^ Due to the exploratory nature of multivariate analyses, the generalisability of the frameworks developed would need to be further tested and validated, and further research with non-migrant workers and workers from other countries would be needed.

## Conclusion

Our study provides a culturally and contextually specific conceptual framework of labour exploitation that gives voice to migrant workers and can be operationalised into a measure of migrant labour exploitation. It calls for the previously overlooked dimension ‘dehumanisation’ and structural forms of coercion to be integrated into mainstream conceptualisations and assessments of labour exploitation to assess its health impacts. Harsh working and employment conditions in London and experiences of sexual abuse revealed in this research call for urgent research and action to improve workplaces and low-paid migrant workers’ safety and health.

## Data Availability

Data are available on reasonable request.
