# Supplementary material for: ‘They treat us like machines’: migrant workers’ conceptual framework of labour exploitation for health research and policy
Source: BMJ Glob Health. 2024 Feb 5;9(2):e013521. doi: 10.1136/bmjgh-2023-013521 (PMC10860016; doi:10.1136/bmjgh-2023-013521)
Supplement: Supplementary data [file bmjgh-2023-013521supp001.pdf]

Additional file

Add. File 1. Distribution of Latin American workers participants' characteristics according to their participation in the phases of the concept mapping

Participants' characteristics	Overall (N=27)		Brainstorming (N=17)		Sorting-rating* (N=23)		Attended both (N=13)	
	n	%	N	%	n	%	n	%
**Female**	11	*40.7*	9	*52.9*	9	*39.1*	7	*53.9*
**Country of birth**								
Colombia	15	*55.6*	9	*52.9*	12	*52.2*	6	*46.2*
Ecuador	7	*25.9*	5	*29.4*	6	*26.1*	4	*30.8*
Other [1]	5	*18.5*	3	*17.7*	5	*21.7*	3	*23.1*
**Level of English**								
fluent or almost	6	*22.2*	3	*17.7*	5	*21.7*	2	*15.4*
can speak but cannot read/write	2	*7.4*	1	*5.9*	2	*8.7*	1	*7.7*
can read/write but cannot speak	6	*22.2*	4	*23.5*	5	*21.7*	3	*23.1*
speak, read/write with difficulty	11	*40.7*	7	*41.2*	11	*47.8*	7	*53.9*
cannot speak, read/write	1	*3.7*	1	*5.9*	0	*0.0*	0	*0.0*
missing	1	*3.7*	1	*5.9*	0	*0.0*	0	*0.0*
**Way s/he found the current job:**								
someone s/he knows told him/her about the job	21	*77.8*	12	*70.6*	20	*87*	1	*84.6*
found it him/herself	3	*11.1*	2	*11.8*	2	*8.7*	1	*7.7*
Other (unemployed)	1	*3.7*	1	*5.9*	0	*0.0*	0	*0.0*
Missing	2	*7.4*	2	*11.8*	1	*4.4*	1	*7.7*
**Type of employer**								
Employed by the workplace where s/he works (in-house / internal employee)	6	*22.2*	2	*11.8*	5	*21.7*	1	*7.7*
employed by an outsourcing company	17	*63*	11	*64.7*	15	*65.22*	9	*69.2*
unemployed	1	*3.7*	1	*5.9*	0	*0.0*	0	*0.0*
Other [2]	2	*7.4*	2	*11.86*	2	*8.7*	2	*15.4*
missing	1	*3.7*	1	*5.9*	1	*4.4*	1	*7.7*
**Current job title**								
cleaner	22	*81.5*	15	*88.2*	18	*78.3*	1	*84.6*
ex-cleaner	1	*3.7*	1	*5.9*	1	*4.4*	1	*7.7*
gardener	1	*3.7*	0	*0.0*	1	*4.4*	0	*0.0*
bartender	1	*3.7*	0	*0.0*	1	*4.4*	0	*0.0*
cook	1	*3.7*	0	*0.0*	1	*4.4*	0	*0.0*
interpreter	*1*	*3.7*	1	*5.9*	1	*4.4*	1	*7.7*
**Highest level of education completed**								
primary school	1	*3.7*	1	*5.98*	1	*4.4*	1	*7.7*
secondary school / A-levels	13	*48.2*	9	*52.9*	11	*47.8*	7	*53.9*
higher education	7	*25.9*	3	*17.7*	7	*30.4*	3	*23.1*
vocational training	2	*7.4*	0	*0.0*	2	*8.7*	0	*0.0*
English certificate	1	*3.7*	1	*5.9*	1	*4.4*	1	*7.7*
missing	3	*11.1*	3	*17.7*	1	*8.7*	1	*7.7*
**Working full-time**								
full-time	13	*48.2*	8	*47.1*	11	*47.8*	6	*46.2*
part-time	13	*48.2*	8	*47.1*	12	*52.2*	7	*53.9*
unemployed	1	*3.7*	1	*5.9*	0	*0.0*	0	*0.0*

Notes: * includes 1 participant who only performed the rating; [1] Spanish-speaking country of central and South America; [2] includes: ‘both in-house and outsourced’; and ‘retired’
